# Laryngopharyngeal reflux image quantization and analysis of its severity

**DOI:** 10.1038/s41598-020-67587-1

**Published:** 2020-07-03

**Authors:** Chung-Feng Jeffrey Kuo, Chih-Hsiang Kao, Sifundvolesihle Dlamini, Shao-Cheng Liu

**Affiliations:** 1 Department of Material Science and Engineering, National Taiwan University of Science and Technology, No. 43, Sec. 4, Keelung Road, Da’an District, Taipei, Taiwan, ROC; 2 Department of Otolaryngology-Head and Neck Surgery, Tri-Service General Hospital, National Defense Medical Center, No. 325, Sec. 2, Cheng-Gong Road, Neihu District, Taipei, 114 Taiwan, ROC

**Keywords:** Computational biology and bioinformatics, Physical sciences, Information technology

## Abstract

Laryngopharyngeal reflux (LPR) is a prevalent disease affecting a high proportion of patients seeking laryngology consultation. Diagnosis is made subjectively based on history, symptoms, and endoscopic assessment. The results depend on the examiner's interpretation of endoscopic images. There are still no consistent objective diagnostic methods. The aim of this study is to use image processing techniques to quantize the laryngeal variation caused by LPR, to judge and analyze its severity. This study proposed methods of screening sharp images automatically from laryngeal endoscopic images and using throat eigen structure for automatic region segmentation. The proposed image compensation improved the illumination problems from the use of laryngoscope lens. Fisher linear discriminant was used to find out features and classification performance while support vector machine was used as the classifier for judging LPR. Evaluation results were 97.16% accuracy, 98.11% sensitivity, and 3.77% false positive rate. To evaluate the severity, quantized data of the laryngeal variation was used. LPR images were combined with reflux symptom index score chart, and severity was graded using a neural network. The results indicated 96.08% accuracy. The experiment indicated that laryngeal variation induced by LPR could be quantized by using image processing techniques to assist in diagnosing and treating LPR.

## Introduction

Laryngopharyngeal reflux (LPR) results from the gastric inclusion flowing back to the throat. It is found that pepsin and gastric acid do major harm to the upper aerodigestive mucosa. Both the laryngeal and pharyngeal mucosa has lower bearing capability for gastric contents^1^. More than 50% of people with voice disorder cases are related to LPR^2^. The primary cause is the upper esophageal sphincter dysfunction^3^. LPR has diverse symptoms, such as the sensation of foreign bodies in the throat, hoarseness, throat clearing, etc. Common signs include mucosal swelling on the larynx and pharynx, subglottic stenosis and vocal edema^4^. It is generally diagnosed by the symptoms and changes in the throat, but there is a lack of convenient and effective objective diagnostic method and if this disease is left untreated it can be one of the etiological causes of laryngeal cancer^5^.

Laryngoscopy is the regular diagnostic method for aerodigestive disorders, but its interpretation is subjective with doubtful efficacy in LPR. According to the reflux finding score (RFS)^6,7^, laryngoscopic examination can be standardized to provide a quantized data, but the inter-rater variability problem exists^8,9^. Detecting pH variations in the throat and the upper end of the esophagus is currently the gold standard^10^. However, this method has restrictions, such as optimal location of the proximal probe, invasiveness and takes 24 h.

The patient’s nonstationary shooting position or lens movement during laryngoscopic examination can lead to nonuniform illumination and blur images. Many studies have used videos, however, the information in the videos were only applicable to functional voice disorders. Therefore, a newer and more objective method is required. The use of image processing has been employed to quantize the changes of the laryngeal mucosa induced by LPR^11^. Jiang et al.^12^ used RGB (red, green, blue) channel values to calculate the red index of various regions of the larynx image. The analysis indicated that the red index of the vocal cords of patients with LPR was much higher. Their study did not discuss texture information. Additionally, the laryngoscope lens was not stationary, leading to inconsistent standard of comparison. The previous researches do not mention how to correct the light source problem resulting from the lens position of the laryngoscope.

Many studies have performed laryngeal sub-region analysis for LPR where the regions were segmented manually which took time and resulted in cognitive differences. Ozturan et al.^13^ analyzed four regions, including the left and right vocal cord, arytenoid cartilage, and epiglottis and choose three points in each region by manual siting. The hues of the points were analyzed to identify LPR. This method is free of the lens position problem and its analysis is rapid, but there are still subjective cognitive differences and the range of the points are only 1 mm in diameter, causing the information to be insufficient.

LPR is treated by lifestyle changes and medication^14,15^, including antacids and proton-pump inhibitors (PPI)^16^. There is still no gold standard for treating LPR, because the examiners cannot specifically judge its severity. Theoretically, the use of image processing to analyze various regions of the larynx is feasible and effective. This study proposed searching for sharp images in laryngeal endoscopic images, in which the larynx was divided by automatic segmentation and the hue and textural features of various regions were analyzed. Our study assumed that LPR could be distinguished more objectively by changes in the hue and texture of various regions of the larynx using support vector machine (SVM). An artificial neural network (ANN) was trained by the quantized data and the reflux symptom index (RSI) score to classify the severity of the LPR.

## Materials and methods

### Data and data acquisition and processing equipment

The image samples were provided by the Department of Otorhinolaryngology-Head and Neck Surgery at Tri-Service General Hospital (Taipei, Taiwan). The subjects were divided into two groups; the non-LPR group composed of 246 subjects including normal patients and those with polyps, cysts and leukamus lesions, and LPR group. The gold standard test for LPR is 24-h pH monitoring, and LPR group comprised of 106 cases that have met all of the following inclusion criteria: The RSI^17^ was higher than 13 points, the total time percentage of the esophagus pH lower than 4 was higher than 1.0%, and the pH test results was abnormal^18^. Excluded cases were patients with anemia, smokers, asthmatic and allergic, patients on medication and patients who has received radiotherapy or neck operations. The images were captured using a laryngeal video stroboscope and processed by MATLAB. Details on the acquisition and processing equipment are provided in the supplementary.

### Selecting the appropriate image

This study proposed screening the sharpest image automatically from dynamic laryngeal endoscopic video using sharp contour of the sharp image. Four functions for judging sharpness were tested: the variance, the sum-modulus-difference, the gradient magnitude maximization and the energy of the Laplacian of the image. See supplementary material for method details. The variance reflects the image’s gray level variation. Among endoscopic images, blurred and distorted images often have blurred contours. The more blurred the contour is, the smaller the variance. The sum-modulus-difference (SMD) uses the first derivative action as a high pass filter to extract the high frequency signals from the image. In terms of the endoscopic image calculation, the gray level difference between two adjacent pixels is calculated and the horizontal (SMD_x_) and vertical (SMD_y_) directions are processed. The sharp image is obtained by calculating the sum of SMD_x_ and SMD_y_, and the endoscope image with the maximum value is selected. The gradient magnitude maximization uses Sobel operation and uses the first derivative to process images. Two masks are used in the endoscopic image for calculation. One is the x-direction and the other is the y-direction. The total gradient is calculated and the endoscopic image in which the maximum value occurs is selected. The energy of the Laplacian of the image uses the second differentiation of the image intensity function as a high pass filter. Laplace’s operation for searching the larynx boundary in the endoscopic image is used for the second differentiation. The approximately discretized Laplace’s operation kernel is used as a high pass filter.

### Automatic segmentation method

Some Refs.^11,13,19,20^ have performed region segmentation of the larynx for follow-up analysis. Pribuišienė et al.^21^ indicated that the most distinctive signs of the LPR are the mucous membrane damage of the true vocal cords and arytenoid cartilage. This region can be displayed completely during shooting to avoid inconsistent feature comparison. This study used automatic segmentation to segment the left and right vocal cords, arytenoid cartilage and glottis. In comparison to manual segmentation, automatic segmentation saves time and is free of subjective cognition.

The glottis is a relatively dark region in the image, therefore the gray threshold was used for segmentation^22^. In order to highlight the differences in brightness, keep more details and avoid over-segmentation, this study calculated the gray level of the full image. The lower bound of the threshold of the image was calculated. This value was taken as binary threshold. Active contour method (ACM)^23,24^ was used since the boundaries of the vocal cords, arytenoid cartilage, and peripheral tissues can be blurred sometimes. Its advantage is that a continuous closed segmentation boundary can be obtained even there is noise. In this method, the target object’s edge detection problem is changed into an energy minimization framework. The purpose is to compute the deformation curve which can minimize the sum of the internal and external energy in an energy field. The internal energy aims to normalize the curve shape and the external energy aims to approach the target object edge and converge to the target object boundary to reconstruct the complete contour of the target object. Let *s* be the parametric curve such that *v*(*s*) = (*x*(*s*), *y*(*s*)), where *x* and *y* are the given curvilinear coordinates; the total energy function *E*_*snake*_ is defined in Eq. (1):

1 $$ E_{snake} = \mathop \smallint \limits_{0}^{1} E_{snake} \left( {v\left( s \right)} \right)ds = \mathop \smallint \limits_{0}^{1} E_{{{\text{int}} ernal}} \left( {v\left( s \right)} \right) + E_{image} \left( {v\left( s \right)} \right) + E_{con} \left( {v\left( s \right)} \right)ds $$

*E*_*internal*_ aims to normalize the stretching and bending of the curve shape and represents the internal energy. *E*_*image*_ is the image energy deduced from the image information, *E*_*con*_ is the constraint energy, and the total energy of *E*_*image*_ and *E*_*con*_ is *E*_*external*_. *E*_*internal*_ is defined as Eq. (2):

2 $$ E_{internal} = \frac{1}{2}\left( {\alpha \left( s \right)\left| {v_{s} \left( s \right)} \right|^{2} + \beta \left( s \right)\left| {v_{ss} \left( s \right)} \right|^{2} } \right), $$

where, |*v*_*s*_(*s*)|^2^ is the curve elasticity, |*v*_*ss*_(*s*)|^2^ is the curvature of the parametric curve, α(*s*) controls the stretching, β(*s*) controls the smootheness of the curve, and *E*_*internal*_ makes the curve contract inward continuously and remain smooth. *E*_*image*_ is defined as Eq. (3):

3 $$ E_{image} = - \left| {\nabla /(x,y)} \right|^{2} , $$

where *I*(*x,y*) is the gradient of image *I* at (*x,y*) and the *E*_*external*_ makes the curve approach the target object contour continuously until they are coincident. *E*_*snake*_ minimization must be coincident, as expressed in Eq. (4):

4 $$ \alpha x^{\prime \prime } (s) - \beta x^{\prime \prime \prime } (s) - \nabla E_{image} = 0. $$

For the physical motion of the snake, *E*_*internal*_ and *E*_*external*_ will be balanced eventually, as expressed in Eq. (5):

5 $$ E_{internal} + E_{external} = \, 0, $$

where $E_{internal} = \alpha x^{\prime \prime } (s) - \beta x^{\prime \prime \prime } (s),$ $E_{external} = - \nabla E_{image}$. The target object can be approached effectively by the continuous initialization of the aforesaid snake model. When ${\text{E}}_{{{\text{snake}}}}$ reaches total energy minimization, the effect is reached. The parametric curve *v*(*s*) is the final contour of object.

### Hue feature

A laryngeal endoscopic image is a color image and is directly applicable to the RGB (red, green, blue) color space. The RGB color space has rapid calculation and does not need to calculate coordinate conversions. Its defect is that the detection result will be affected by the environment and light source. In order to circumvent this issue, this study propose the use of two color spaces which are free from the effect of brightness: the HSV (hue, saturation, value) color space and the YC_b_C_r_ color space^25^. In the HSV color space, H = 0° represents red, H = 120° represents green and H = 240° represents blue. S is 0–1, and the image is a gray level image when S = 0. V represents brightness, in which V = 0 represents black and V = 1 represents white. The main components of the YC_b_C_r_ color space are the brightness (Y) and two chromaticies (C_b_, C_r_). Y is the gray level of the gray scale image converted from a color image. The brightness separability is very high and is favorable for adjusting different chromaticity components. C_b_ is the blue chromaticity component and C_r_ is the red chromaticity component. The YC_b_C_r_ color space can reduce the effect of brightness, hence it is used in image processing techniques. This study used chromaticity as a hue feature, and there were six chromatic values (R-G-B-H-C_b_-C_r_) used as hue features. The left and right vocal cord, arytenoid cartilage, and the ratio of the vocal cords to arytenoid cartilage were analyzed. There were four region analysis with 24 hue features in all.

### Textual features

In terms of the perceptual experience of the human eye, the rough and directionality are the primary characteristics used by the human eye to distinguish texture. The Gray-level Co-occurrence Matrix (GLCM)^26,27^ describes the grayness relationship between adjacent pixels in a local area or overall area of an image. To quantize the laryngeal variation induced by LPR, this study used the equalization, contrast, correlation and homogeneity of GLCM to describe the texture information of various regions of larynx. The angle was set as 0° to analyze the features of LPR. Normalization was performed before the GLCM eigenvalue were extracted and the sum of the elements of GLCM was set as 1 for computing. The eigenvalues used are discussed below.

#### Equalization (***E***)^28^

This eigenvalue is known as the energy, which was used in this study to measure the consistency and equalization of the gray level distribution in each region of an image. Consistency and equalization refer to the probability of the occurrence of a pixel pair and a higher probability of recurrence represents higher consistency and equalization. The range of (*E*) of GLCM was [0, 1]. It reaches the maximum value (*E* = 1) when the gray levels of the image were identical.

#### Contrast (***Con***)^28^

This eigenvalue was used to measure the intensity contrast between adjacent pixels in each region of the image. A larger gray level difference between adjacent pixels represent a larger *Con* value of GLCM. In a *k *×* k* GLCM, the range of *Con* would be [0, (k − 1)^2^].

#### The correlation (*Cor*)

This one was used to measure the correlation between adjacent pixels in each region.

#### Homogeneity (*Hom*)

It was used to measure the local grayness homogeneity in each region. If the local grayness homogeneity of the image was uniform, the *Hom* value would be large.

### Classifiers

The features of various regions were extracted and classified to identify LPR. SVM^29^ has a good training and classification execution speed and will separate the hyperplane during computing. This study extracted the hue and textural features of various regions of the larynx to accurately identify LPR. SVM was effective for dividing samples into negative and positive. An ANN has strong nonlinear fitting capability, strong noisy data tolerance and is characterized by multiple entry/exit features. The LPR severity analysis required multiple classification results and the ANN met the requirements of this study for strong error tolerance and multiple entry/exit characteristics. SVM^30,31^ is a binary linear classifier that is used to find a hyper-plane in a space so that two classes of data can be separated. It is used to find a zone with the maximum boundary in two different classes of data. The hyper-plane is called the optimal hyper-plane and the class of the unknown data is determined according to the data position in it. The back-propagation neural network (BPNN)^32–34^ has a forward recurrent learning ability network. This study used BPNN as a classifier to analyze the LPR severity because of its higher error tolerance and multiple entry/exit characteristics. The architecture was comprised of three layers; input layer, hidden layer and output layer as shown in supplementary Fig. 1.

## Results and validation

**Figure 1:**
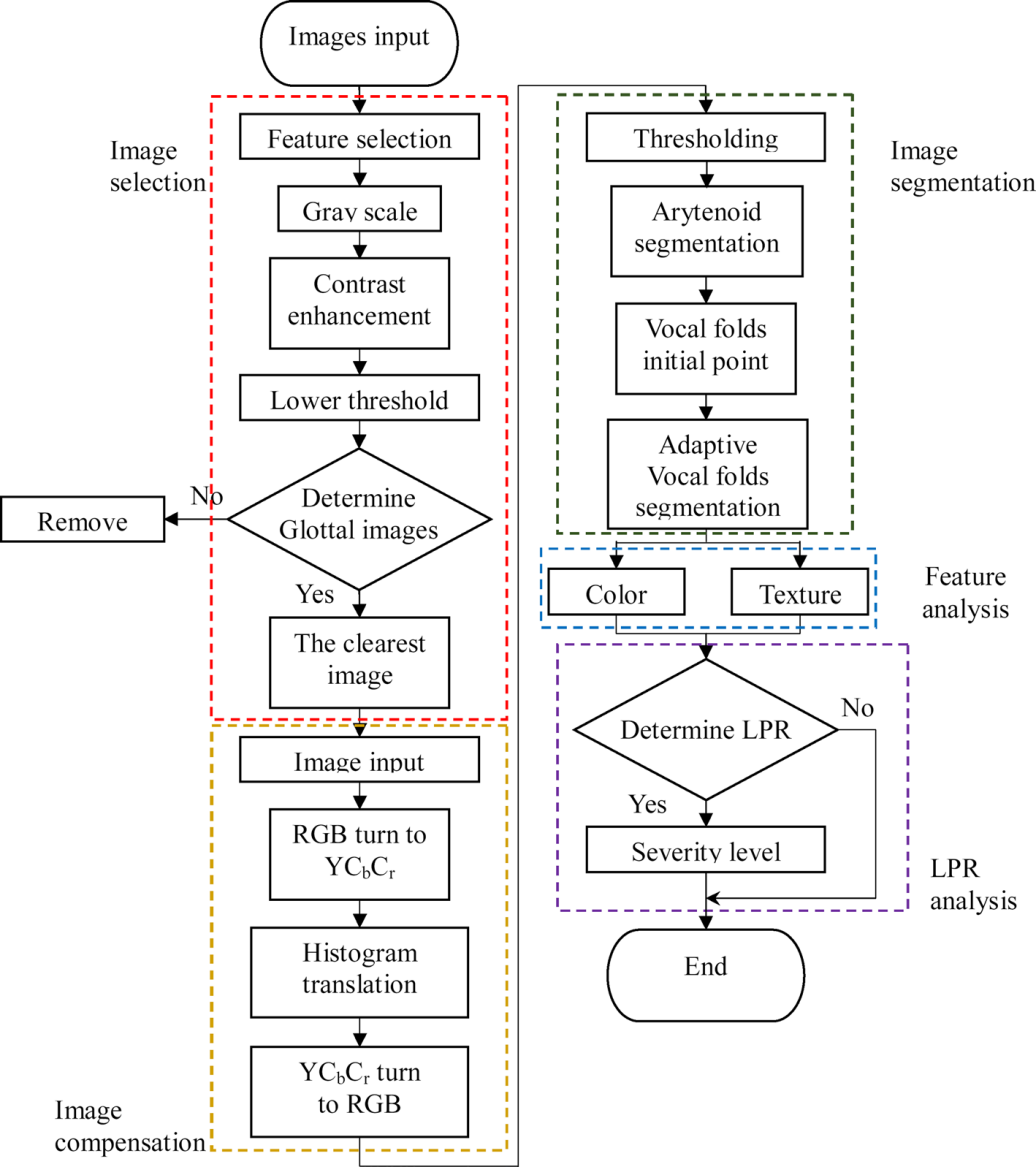
System flow chart.

Clear laryngeal endoscopic images were searched for automatic segmentation and feature analysis. The automatically-segmented regions included left and right vocal cords, and the arytenoid cartilage, and the hue and textural features of various regions were analyzed. LPR can be diagnosed by using image processing techniques to analyze larynx image features. This study proposed using the quantized data of variations of the throat induced by LPR to analyze its severity and provide doctors medical advice and assistance with patient diagnosis. The detection system was divided into five parts as shown in Fig. 1.

### Image selection

#### Feature filtration

Non-throat images were filtered using red component. Blurred images were eliminated using the rapid execution of variance. Images with a red component less than 0.4 were filtered out. The average value of variance of the remaining images was calculated and images with a lower than average value of the variance were removed.

#### Glottis structure discrimination

**Figure 2:**
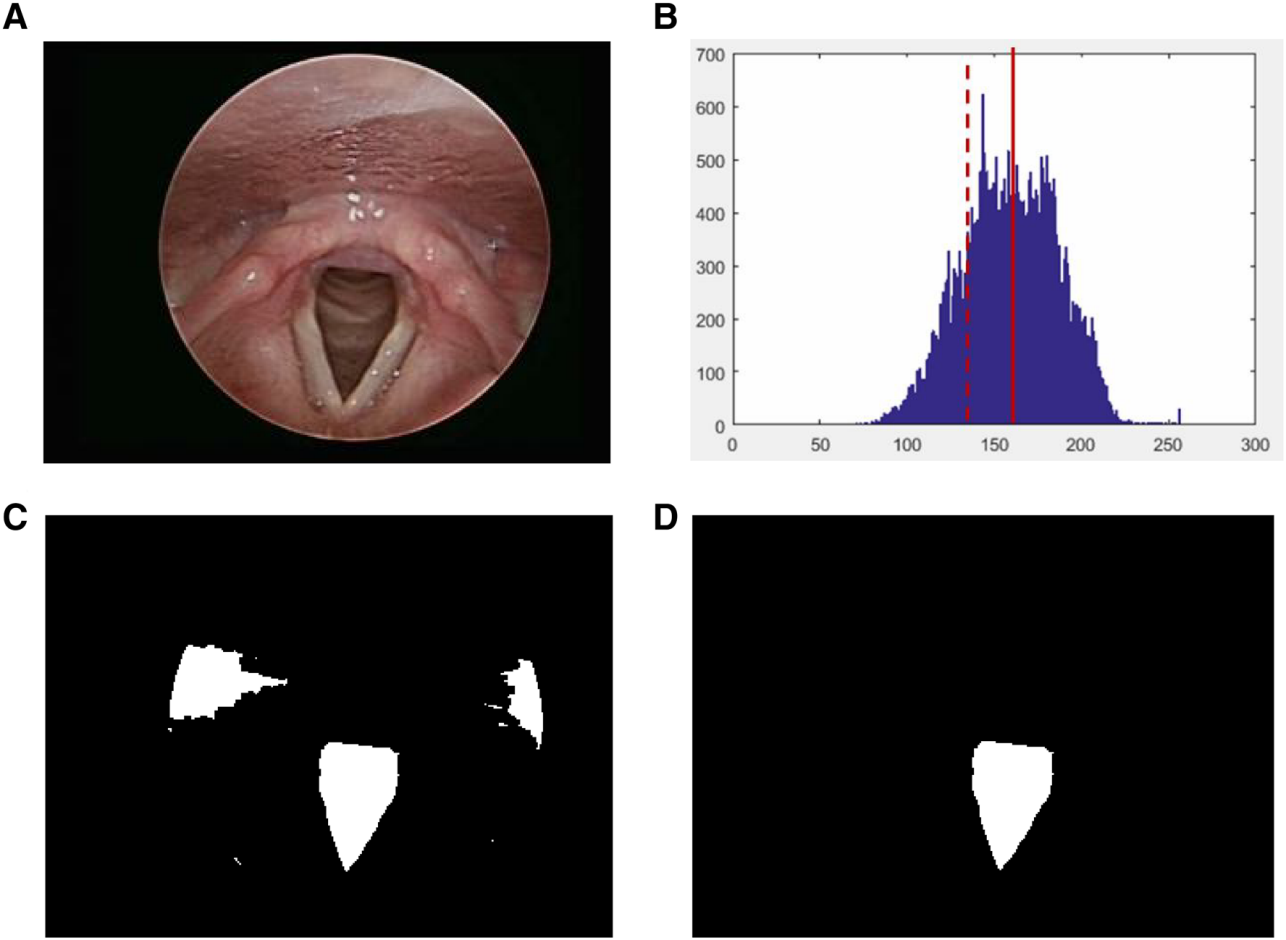
Glottis segmentation process: (**A**) original image, (**B**) gray level histogram of original image [the solid red line is the average value (u = 158) and the dotted red line is the lower bound of the threshold (TH = 131)], (**C**) binary image, (**D**) glottis discrimination.

The glottis is a relatively dark region in the image, as shown in Fig. 2A. The lower bound of threshold is used as the image binarization, as shown in Fig. 2B. The binary image is shown in Fig. 2C. This study used glottis structure condition for screening. The conditions were: area, centroid position and aspect ratio. Regions that were too small were excluded, regions where the glottis was not in the center were eliminated, and since glottis shape is an inverted triangle, the east–west regions were filtered out using aspect ratio. The glottis segmentation is shown in Fig. 2D.

#### Sharp image

**Figure 3:**
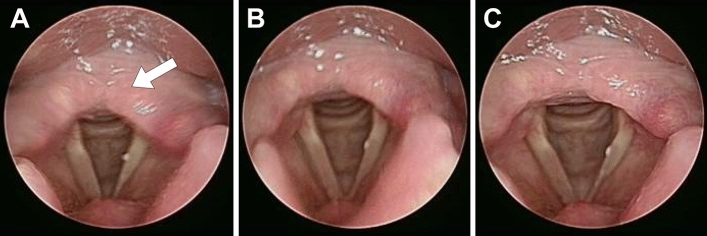
(**A**) The shift of arytenoid cartilage results in apparent blur (arrowhead), (**B**) unfocused lens results in blurred image, (**C**) sharp image.

**Figure 4:**
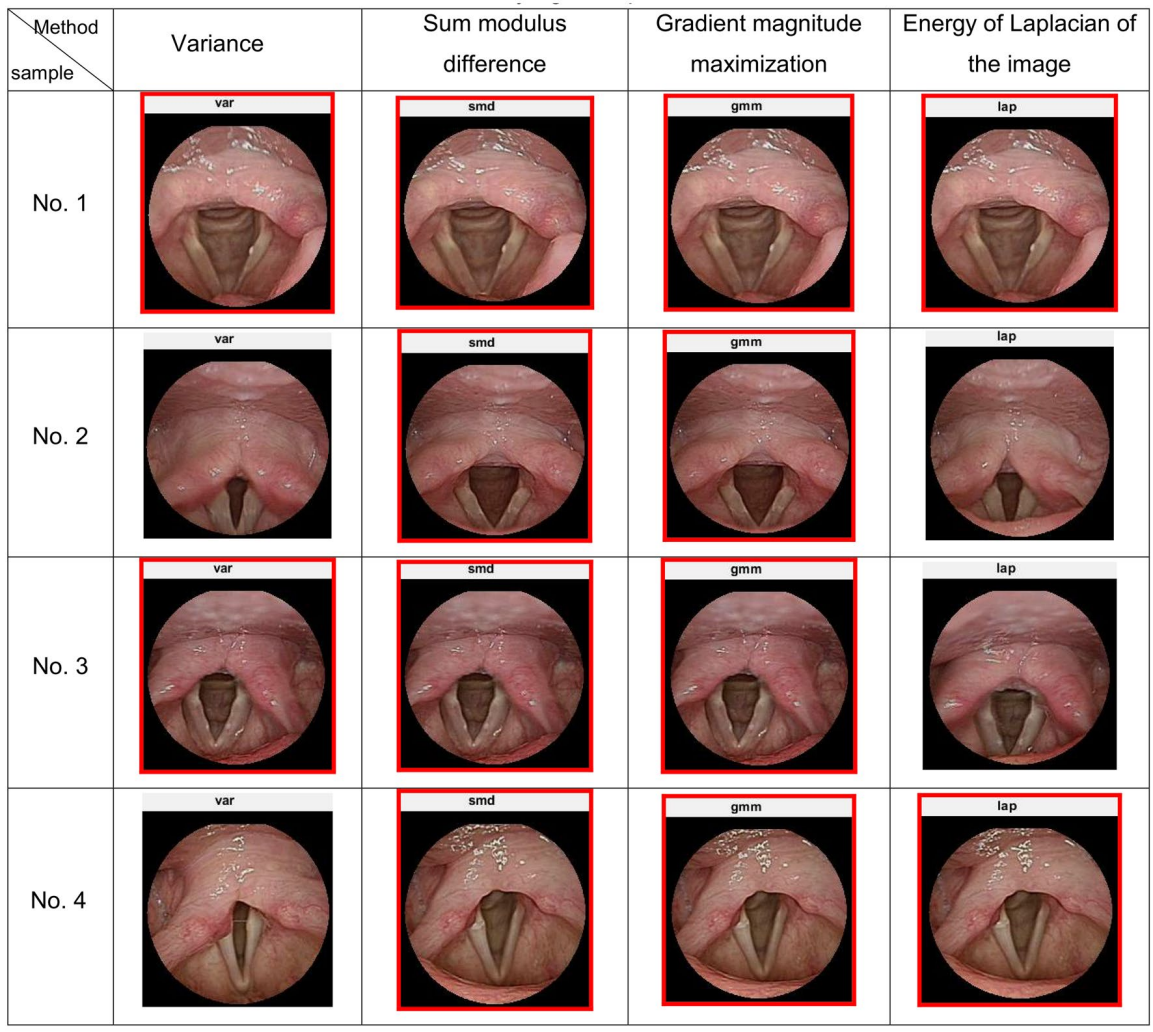
Test of the four methods to judge sharpness.

After the glottis is screened out, the sharp image is screened out of multiple images, because most images are blurred as the patient or lens moves, i.e. insufficient sharpness. The object boundary in the blurred image is quite blurred; on the contrary, the object boundary in the sharp image is relatively clear, as shown in Fig. 3. The Fig. 3A image is obviously blurred due to the movement of arytenoid cartilage (arrowhead); the Fig. 3B is a blurred image as the lens is unfocused; Fig. 3C is a sharp image. This study tested and compared 10 sample and used four functions for judging the sharpness: the variance, the sum-modulus-difference, the gradient magnitude maximization, and the energy of the Laplacian of the image. It is obvious that the red frame images are relatively sharp. This study used sum-modulus-difference to search for the sharpest throat image, as this method had better execution speed and good precision as shown in Fig. 4.

### Image compensation

**Figure 5:**
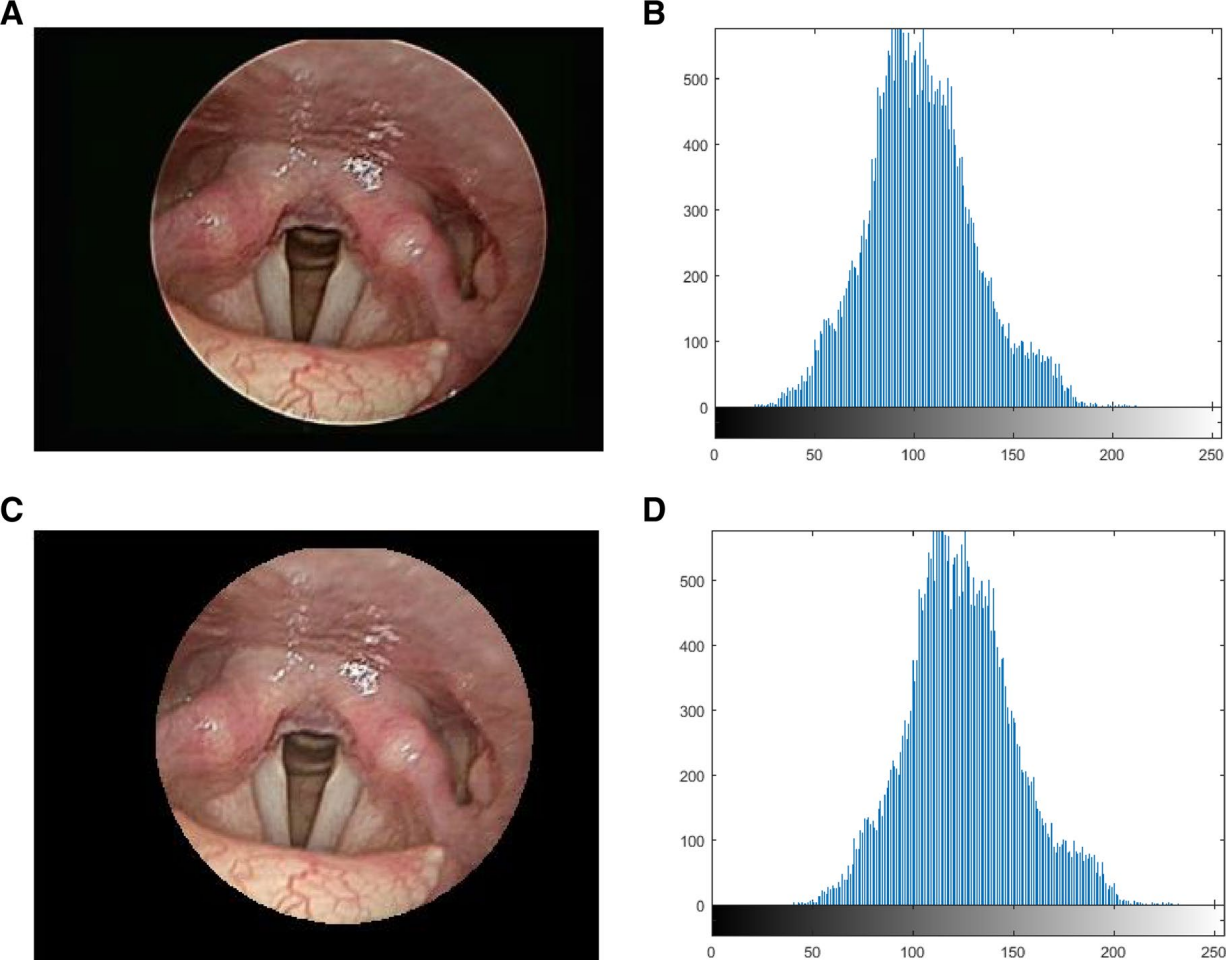
Image compensation: (**A**) original image, (**B**) brightness histogram of the original image (average: 103.72), (**C**) image compensation, (**D**) brightness histogram of the image compensation (average: 125).

In order to allow similar color ranges of the laryngeal endoscopic images, histogram shifting was used to unify the average gray level of all images to the same value. Based on the difference between the average brightness value of the throat image and the set value, each pixel was increased or decreased to achieve the set average value. The formula is converted as follows: I(x,y) = O(x,y) + (S – Om), where (x, y) is the image coordinate, I is the grayscale value of the image after translation, O is the grayscale value of the current image, S is the set value, and Om is the grayscale value of the image before translation. The RGB was turned into YC_b_C_r_ space where the Y channel value was shifted to 125. The image was turned back to RGB space to enable consistent standard for the subsequent hue features. The proposed method could avoid the light source problem resulting from the laryngoscope lens position and allow the segmentation to be more correct. Figure 5 shows the image compensation.

### Region segmentation

#### Arytenoid cartilage segmentation

The image was binarized, relatively bright regions including the arytenoid cartilage were turned into white and the binary image was morphologically eroded. The largest region obtained by the notation was regarded as the arytenoid cartilage candidate block and discrimination of the arytenoid cartilage structure was performed. Finally, the region of arytenoid cartilage was obtained using ACM. The study then used the arytenoid cartilage structure conditions for screening which were: area, centroid position and aspect ratio. Regions that were too small were eliminated, regions where the arytenoid cartilage was not in the upper part were eliminated, and since the arytenoid cartilage shape is rectangular, the south-north regions were filtered out using aspect ratio. The arytenoid cartilage segmentation is shown in Supplementary Fig. 2.

#### Vocal cord segmentation

Both the vocal cords were presented as columnar features near the glottis and appear relatively brighter. This study used the vocal cord position for segmentation. First, the glottis was morphologically dilated and the edge image was taken. The glottis was transected using its centroid, and the valley was searched and slit as shown in Supplementary Fig. 3.

#### Adaptive vocal cord segmentation

**Figure 6:**
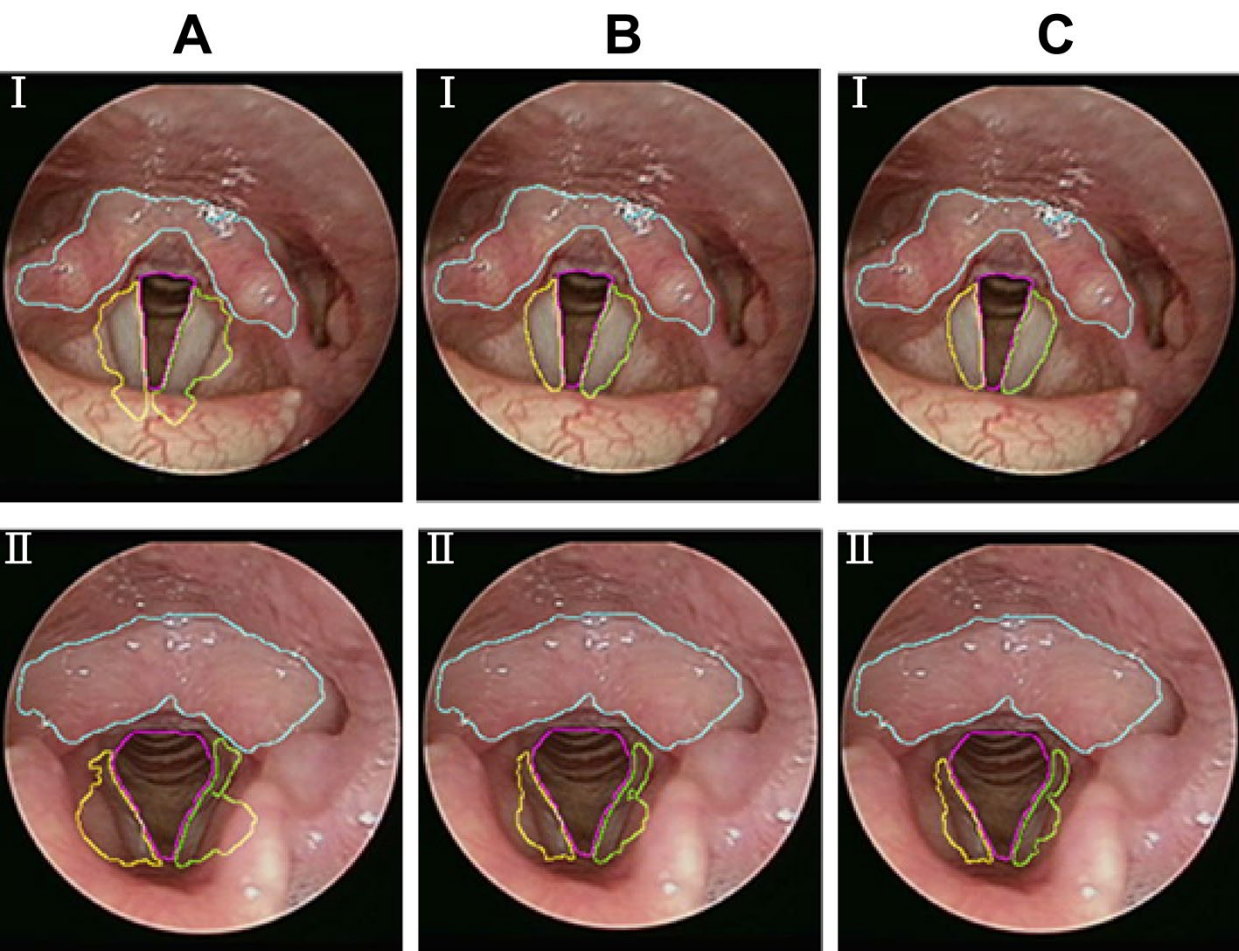
Left vocal cord and right vocal cord of Sample I and Sample II iterated the same number of times: (**A**) 41 iterations, (**B**) 25 iterations, (**C**) 20 iterations.

Laryngeal endoscopic images do not have a fixed lens distance, therefore the vocal cord size varies, which means the number of iterations for the ACM cannot be fixed as shown in Fig. 6. In addition, since the left and right vocal cord size could differ, there would be obvious over-segmentation or under-segmentation under a fixed value. In order to solve this, the study proposed an adaptive method to determine the number of iterations. The growth range of the ACM was mitigated gradually as the gap between the vocal cords and the false vocal cords narrowed. The difference between the gray level variations in the range grown by this iteration and in the growth range of the last iteration was slight and could be described by calculating the entropy of the gray level variation. This is to say, when the difference in the gray level and entropy in the range grown by two iterations were slight, the entropy will have a minimum difference. If the growth range increased continuously, the growth range would exceed the vocal cord and the entropy difference between the range grown by this iteration and the growth range of the last iteration would increase. Using this characteristic, the entropy of the growth range was calculated during every iteration and subtracted from the last iteration to find out the number of iterations with the minimum entropy difference. The result was the optimum number of iterations of the vocal cord. The minimum number of iterations of the ACM was 13, the maximum was 41, and the entropy of the growth range of every iteration was calculated. The entropy difference between iterations was calculated and the minimum difference was found out. In this example, the minimum entropy was 21st iteration and the entropy difference was 0.0043. As the minimum number of iterations of the ACM in this study was 13, the minimum entropy difference was the 21st iteration, meaning the growth ranges of the 33rd, 34th iterations of the ACM were approximately saturated. The optimum number of iterations of the left and right vocal cord could be found out as shown in Supplementary Fig. 4.

#### Segmentation accuracy validation

In order to determine the segmentation accuracy, dice similarity coefficient (DSC)^35,36^ was used to calculate the segmentation accuracy corresponding to each sample. This study drew 10 samples randomly and the manual segmentation performed by three doctors was compared with the method proposed in this study refer to Table 1 showing the DSC average value of the four regions for the 10 samples in the supplementary. The DSC of the four regions were 0.8674 for the left vocal cord, 0.8460 for the right vocal cord, 0.8796 for the arytenoid cartilage, and 0.9293 for the glottis. The results proved that the proposed automatic segmentation was accurate and stable.

**Table 1:** DSC average value of the four regions of the 10 samples.

Sample	Region			
	Arytenoid cartilage	Left vocal cord	Right vocal cord	Glottis
No. 1	0.9106	0.8073	0.7643	0.8773
No. 2	0.9053	0.8843	0.8935	0.9234
No. 3	0.9288	0.8210	0.7939	0.9455
No. 4	0.8981	0.8150	0.8084	0.9394
No. 5	0.8361	0.8999	0.8095	0.9166
No. 6	0.8405	0.9163	0.8999	0.9454
No. 7	0.8847	0.9185	0.8576	0.9490
No. 8	0.8750	0.9012	0.8965	0.9213
No. 9	0.8601	0.8697	0.8352	0.9440
No. 10	0.8563	0.8411	0.9010	0.9311
Average	0.8796	0.8674	0.8460	0.9293

### Feature selection

In order to detect LPR, different features of various regions were required, therefore hue and textural features were used. This study performed the Fisher linear discriminant of 76 LPR and non-LPR samples. 36 features of different color spaces and textures in four regions were tested and the Fisher linear discriminator was used to find out the features with classification performance for LPR. It was found that the C_b_ channel and C_r_ of the arytenoid cartilage, the R channel of the vocal cords, the energy of GLCM of the arytenoid cartilage, and the contrasted Fisher linear discriminant of the vocal cord GLCM were 0.6425, 0.6409, 0.5213, 0.6568, and 0.5241 respectively representing the highest among the 36 features. Table 2 shows the classification capacity of various features.

**Table 2:** Feature calculation for the Fisher linear discriminant.

Feature	*f*	Feature	*f*
A_R	0.0177	VC/A_R	0.2836
A_G	0.0373	VC/A_G	0.0575
A_B	0.0226	VC/A_B	0.0708
VC_R max	**0.5213**	VC/A_H	0.0327
VC_R min	0.3015	VC/A_Cb	0.3671
VC_G max	0.0021	VC/A_Cr	0.2012
VC_G min	0.0081	A_con	0.2378
VC_B max	0.2172	A_eng	**0.6568**
VC_B min	0.0984	A_cor	0.0087
A_H	0.0629	A_hom	0.2052
VC_H max	0.0126	VC_con max	**0.5241**
VC_H min	0.0022	VC_con min	0.22
A_Cb	**0.6425**	VC_eng max	0.018
VC_Cb max	0.0317	VC_eng min	0.0236
VC_Cb min	0.0061	VC_cor max	0.0019
A_Cr	**0.6409**	VC_cor max	0.0061
VC_Cr max	0.0411	VC_hom min	0.038
VC_Cr min	0.0433	VC_hom max	0.0183

Values highlighted in bold represent significant difference among the 36 features

### LPR analysis

In order to diagnose LPR, this study used SVM as a classifier. There were 352 research samples, including 106 LPR and 246 non-LPR samples. According to Choi and Choi^37^ and Javaid et al.^38^, when the sample ratio is unbalanced, the positive–negative sample ratio of 1:1 is recommended for K = 10 cross validation during classification evaluation. Therefore, this study used 106 LPR and non-LPR samples for validation, and the results of the classification was evaluated 10 times using cross validation.

#### LPR identification results analysis

The non-LPR samples were defined as positives and the LPR samples were defined as negative. The results showed that both LPR and non-LPR are True. Table 3 shows the classification results. This study used accuracy, sensitivity, and false positive rate to evaluate the classification results.

**Table 3:** LPR classification accuracy.

Accuracy	Sensitivity	False positive rate
97.16%	98.11%	3.77%

#### Severity classification

The severity was preliminarily classified into three levels according to the classification of the digestant, which was combined with RSI as the classification criteria. RSI scores of 13–20 were primary, 21–30 were intermediate, and 31–45 were severe. There were 106 samples in this study, including 38 primary samples, 54 intermediate, and 14 severe samples. Stratified cross validation was used for classification, and tenfold stratified cross validation was used to evaluate the results of classification. The BPNN architecture was divided into the input, hidden, and output layers. The input layer had five processing units representing five eigenvalues, respectively which were the C_b_ and C_r_ channels of the arytenoid cartilage, the energy of GLCM, the R channel of the vocal cords and the contrast of the GLCM. There was one hidden layer and four processing units, and the output layer judge the LPR. The parameters of the BPNN used in this study were tested continuously through trial and error. The final cycle index was 1,000 times, the learning rate was 0.65 and the momentum factor was 0.5. Stratified cross validation was performed for this group of parameters to evaluate the results of classification and the overall recognition rate was 96.48% as show in Table 4.

**Table 4:** LPR severity classification results.

Cross validation	Accuracy (%)
No. 1	96.13
No. 2	97.82
No. 3	98.64
No. 4	94.32
No. 5	95.47
No. 6	97.77
No. 7	96.32
No. 8	95.61
No. 9	97.78
No. 10	94.36
Average accuracy	96.48

## Discussion

Manual examination of LPR using a laryngoscope is subjective. In this case, giving treatment according to the symptoms without specific diagnostic evidence may result in medical and economic burdens^38^. Using image processing techniques to analyze the hue and texture of the laryngeal images is a more objective technique. In order to identify LPR accuracy, obtaining sharp images and uniform light source is a priority for analyzing images. In the study, the sharpest larynx image was found out by using the variance and the sum-modulus-difference in laryngoscopic images. The image compensation proposed in this study used histogram shifting to give a consistent brightness range and to prevent the gray level of the image from exceeding boundaries that failed to display.

The most distinctive sign of LPR is the mucosal damage on the true vocal cords and the arytenoid cartilage. In this study, the arytenoid cartilage, glottis, left and right vocal cords were segmented automatically for analysis. This study used DSC for segmentation validation and the results proved that the proposed automatic segmentation was accurate and stable. Du et al.^19^ and Witt et al.^20^ mentioned changes in hue and texture but did not indicate the regions in which hues and which textures are more distinctive for LPR. This study tested different color spaces and textures of four regions (36 features) and used the Fisher linear discriminant to find the features with classification performance for LPR. It was found that the C_b_ and the C_r_ channels of the arytenoid cartilage, the R channel of the vocal cords, the energy of GLCM of the arytenoid cartilage, and the contrasted Fisher linear discriminant of the vocal cord GLCM were outstanding. Our results revealed that LPR could induce changes in the larynx which cannot be described by human eye specifically. The aforementioned five features were combined with SVM to distinguish LPR and non-LPR conditions. The LPR recognition accuracy of the proposed method was 97.16%, the sensitivity was 98.11% and the false positive rate was 3.77%, proving that LPR could be identified according to the hue and textural features.

This study used the quantized data of laryngeal variation induced by LPR and RSI as the training and output samples of the BPNN. The five features and RSI were used as training samples of the BPNN and the overall recognition rate was 96.48%. The RSI evaluation method was subjective, but large amount of RSI information approached the objective results. The test results of the RSI samples indicated that the severity of LPR could be classified by quantized data of the laryngeal variations induced by LPR. The results could be used by doctors to provide medication suggestion for patients in real-time treatment.

## Conclusion

This study proposed searching for sharp larynx images in videos taken by a laryngoscope, to solve the difficulty in capturing sharp images. In order to eliminate the light source problem resulting from an inconsistent laryngoscope lens position, histogram shifting was used to give samples a consistent gray level range for subsequent region segmentation and feature analysis. The automatic segmentation of the larynx segments was consistent across all samples, the subjective differences of the manual segmentation were reduced, and the manual augmentation time was saved. The five features with discriminability for LPR were combined with SVM, and the LPR recognition result had high precision. In terms of severity of LPR, the five features were combined with RSI for ANN training. The results showed that using the quantized data of LPR images to classify the severity could assist doctors in diagnosis.

### Ethics approval

The research protocol (No.: 1-108-05-132) has been reviewed and approved by the Institutional Review Board of Tri-Service General Hospital.

### Consent for publication

All methods were performed in accordance with the relevant guidelines and regulations. All patients provided written informed consent prior to participation.

## Supplementary information

Supplementary file1 (DOC 1258 kb)

## Data Availability

The datasets generated from this study are available from the corresponding author on reasonable request.

## References

[1] Koufman JA, Aviv JE, Casiano RR, Shaw GY (2002). Laryngopharyngeal reflux: position statement of the committee on speech, voice, and swallowing disorders of the American Academy of Otolaryngology-Head and Neck Surgery. Otolaryngol. Head Neck Surg..

[2] Mhabish FM, Al Yasiri R (2017). Aetiology of hoarseness in patients above 40 years of age. Al-Qadisiah Med. J..

[3] Postma GN, Belafsky PC, Tomek MS, Koufman JA (2001). Esophageal motor function in laryngopharyngeal reflux is superior to that in classic gastroesophageal reflux disease. Ann. Otol. Rhinol. Laryngol..

[4] Powell J, Cocks HC (2013). Mucosal changes in laryngopharyngeal reflux—prevalence, sensitivity, specificity and assessment. Laryngoscope.

[5] Salihefendic N, Zildzic M, Cabric E (2017). Laryngopharyngeal reflux disease–LPRD. Med. Arch..

[6] Belafsky PC, Postma GN, Koufman JA (2001). Laryngopharyngeal reflux symptoms improve before changes in physical findings. Laryngoscope.

[7] Belafsky PC, Postma GN, Koufman JA (2001). The validity and reliability of the reflux finding score (RFS). Laryngoscope.

[8] Cheng F-KF, Albert DM, Maydonovitch CL, Wong RK, Moawad FJ (2015). Categorization of patients with reflux symptoms referred for pH and impedance testing while off therapy. Clin. Gastroenterol. Hepatol..

[9] Kim S (2017). Association between 24-hour combined multichannel intraluminal impedance-pH monitoring and symptoms or quality of life in patients with laryngopharyngeal reflux. Clin. Otolaryngol..

[10] Sakin YS (2017). The diagnostic value of 24-hour ambulatory intraesophageal pH-impedance in patients with laryngopharyngeal reflux symptoms comparable with typical symptoms. United Eur. Gastroenterol. J..

[11] Nayak A, Kumar S, Arora R, Singh GB (2018). Image analysis of interarytenoid area to detect cases of laryngopharyngeal reflux: an objective method. Am. J. Otolaryngol..

[12] Jiang J, Chi W (1998). Quantitative color analysis of laryngeal erythemain chronic posterior laryngitis. J. Voice.

[13] Ozturan O, Dogan R, Yenigun A, Veyseller B, Yildirim YS (2017). Photographic objective alterations for laryngopharyngeal reflux diagnosis. J. Voice.

[14] Guo H, Ma H, Wang J (2016). Proton pump inhibitor therapy for the treatment of laryngopharyngeal reflux. J. Clin. Gastroenterol..

[15] Lin RJ, Sridharan S, Smith LJ, Young VN, Rosen CA (2018). Weaning of proton pump inhibitors in patients with suspected laryngopharyngeal reflux disease. Laryngoscope.

[16] Lazarus B (2016). Proton pump inhibitor use and the risk of chronic kidney disease. JAMA Int. Med..

[17] Belafsky PC, Postma GN, Koufman JA (2002). Validity and reliability of the reflux symptom index (RSI). J. Voice.

[18] Muderris T, Gokcan MK, Yorulmaz I (2009). The clinical value of pharyngeal pH monitoring using a double-probe, triple-sensor catheter in patients with laryngopharyngeal reflux. Arch. Otolaryngol. Head Neck Surg..

[19] Du C, Ramahi J, Liu Q, Yan Y, Jiang J (2017). Validation of the laryngopharyngeal reflux color and texture recognition compared to pH-probe monitoring. Laryngoscope.

[20] Witt DR (2014). Detection of chronic laryngitis due to laryngopharyngeal reflux using color and texture analysis of laryngoscopic images. J. Voice.

[21] Pribuišienė R, Uloza V, Kupčinskas L (2008). Diagnostic sensitivity and specificity of laryngoscopic signs of reflux laryngitis. Medicina.

[22] Al-Amri, S. S. & Kalyankar, N. V. Image segmentation by using threshold techniques. *arXiv preprint arXiv:1005.4020* (2010).

[23] Rebouças Filho PP, Cortez PC, da Silva Barros AC, Albuquerque VHC, Tavares JMR (2017). Novel and powerful 3D adaptive crisp active contour method applied in the segmentation of CT lung images. Med. Image Anal..

[24] Rebouças Filho PP, Cortez PC, da Silva Barros AC, De Albuquerque VHC (2014). Novel Adaptive Balloon Active Contour Method based on internal force for image segmentation—a systematic evaluation on synthetic and real images. Expert Syst. Appl..

[25] Shaik KB, Ganesan P, Kalist V, Sathish B, Jenitha JMM (2015). Comparative study of skin color detection and segmentation in HSV and YCbCr color space. Proc. Comput. Sci..

[26] Kuffer M, Pfeffer K, Sliuzas R, Baud I (2016). Extraction of slum areas from VHR imagery using GLCM variance. IEEE J. Select. Top. Appl. Earth Obs. Remote Sens..

[27] Albà X (2018). Automatic initialization and quality control of large-scale cardiac MRI segmentations. Med. Image Anal..

[28] Lloyd K, Rosin PL, Marshall D, Moore SC (2017). Detecting violent and abnormal crowd activity using temporal analysis of grey level co-occurrence matrix (GLCM)-based texture measures. Mach. Vis. Appl..

[29] Ramírez J (2013). Computer-aided diagnosis of Alzheimer’s type dementia combining support vector machines and discriminant set of features. Inf. Sci..

[30] Peruzzo D (2016). A framework for the automatic detection and characterization of brain malformations: validation on the corpus callosum. Med. Image Anal..

[31] Wang Z (2017). Multi-modal classification of neurodegenerative disease by progressive graph-based transductive learning. Med. Image Anal..

[32] Messay T, Hardie RC, Tuinstra TR (2015). Segmentation of pulmonary nodules in computed tomography using a regression neural network approach and its application to the lung image database consortium and image database resource initiative dataset. Med. Image Anal..

[33] Wang S (2017). Central focused convolutional neural networks: Developing a data-driven model for lung nodule segmentation. Med. Image Anal..

[34] Leung H, Haykin S (1991). The complex backpropagation algorithm. IEEE Trans. Signal Process..

[35] Dice LR (1945). Measures of the amount of ecologic association between species. Ecology.

[36] Lê M, Unkelbach J, Ayache N, Delingette H (2016). Sampling image segmentations for uncertainty quantification. Med. Image Anal..

[37] Choi W-J, Choi T-S (2014). Automated pulmonary nodule detection based on three-dimensional shape-based feature descriptor. Comput. Methods Programs Biomed..

[38] Javaid M, Javid M, Rehman MZU, Shah SIA (2016). A novel approach to CAD system for the detection of lung nodules in CT images. Comput. Methods Programs Biomed..

