# Supplementary material for: Laryngopharyngeal reflux image quantization and analysis of its severity
Source: Sci Rep. 2020 Jul 3;10:10975. doi: 10.1038/s41598-020-67587-1 (PMC7335083; doi:10.1038/s41598-020-67587-1)

Title: Laryngopharyngeal reflux image quantization and analysis of its severity

Chung-Feng Jeffrey Kuo^1^, Chih-Hsiang Kao^1^, Sifundvolesihle Dlamini^1^, Shao-Cheng Liu^2*^

*^1^Department of Material Science & Engineering, National Taiwan University of Science and Technology, Taipei, Taiwan, Republic of China*

*^2^Department of Otolaryngology-Head and Neck Surgery Tri-Service General Hospital, National Defense Medical Center, Taipei, Taiwan, Republic of China*

**^＊^**Corresponding author:

Shao-Cheng Liu, M.D. PhD.

Associate Professor

Department of Otolaryngology-Head and Neck Surgery,

Tri-Service General Hospital, National Defense Medical Center,

No. 325, Sec. 2, Cheng-Gong Road, Neihu District, Taipei, Taiwan 114, R.O.C.

Tel: 886-2-8792-7192

Fax: 886-2-8792-7193

Shao-Cheng Liu, M.D. PhD.

Associate Professor

Department of Otolaryngology-Head and Neck Surgery,

Tri-Service General Hospital, National Defense Medical Center,

No. 325, Sec. 2, Cheng-Gong Road, Neihu District, Taipei, Taiwan 114, R.O.C.

Tel: 886-2-8792-7192

Fax: 886-2-8792-7193

E-mail: E-mail: [m871435@ndmctsgh.edu.tw](mailto:m871435@ndmctsgh.edu.tw)

Acknowledgments

The research was supported by Tri-Service General Hospital, National Defense Medical Center-National Taiwan University of Science and Technology Joint Research Program (TSGH-C109-010, TSGH-NTUST-109-02).

**Details on the acquisition and processing equipment**

A. Computer

1. Operating system: Microsoft Windows 7 64-bit
2. CPU: Athlon(tm) II X4 640 Processor 3.0 GHz
3. Memory: 12.00 GB RAM

B. Software

1. Programming language: MATLAB 2014b

C. Image capture device

1. Laryngeal video stroboscope: a Karl storz 8706CA from Krl storz Company and KAyPentax 9106 from KayPentax
2. Light source: Olympus Visera elite CLV-S190
3. Processor: Plympus Visera elite OTV-S190

**Variance**
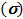
**.** The variance reflects the image’s gray level variation. Among endoscopic images, blurred and distorted images often have blurred contours. The more blurred the contour is, the smaller the variance, as expressed in Eq. (1):

| 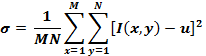 | (1) |
| --- | --- |

where, *M* and *N* are the image size, *I* is the image, *x* and *y* are the pixel position, and *u* is the average value of the image.

**Sum-modulus-difference**

The sum-modulus-difference uses the first derivative action as a high pass filter to extract the high frequency signals from the image. In terms of the endoscopic image calculation, the gray level difference between two adjacent pixels is calculated and the horizontal
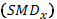
 and vertical
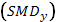
 directions are processed. The deviation coefficient of
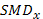
 and
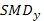
 is calculated as Eqs. (2) and (3) respectively:


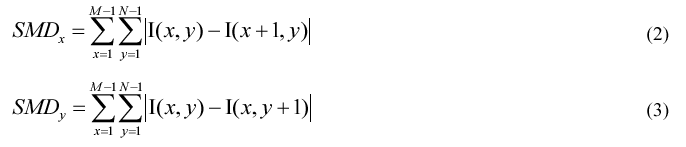


where, *M* and *N* are the image size, *I* is the image, and *x* and *y* are the pixel position.

The sharp image is obtained by calculating the sum of
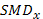
 and
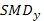
, and the endoscope image with the maximum value is selected as expressed in Eq. (4):

| 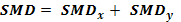 | (4) |
| --- | --- |

**Gradient magnitude maximization**

The Gradient magnitude maximization uses Sobel operation, and uses the first derivative to process images. Two masks are used in the endoscope image for calculation. One is x-direction, the other one is y-direction, expressed as Eqs. (5) and (6).

| $i_{x}=\frac{1}{4}\left[\begin{matrix} -1 & 0 & 1 \\ -2 & 0 & 2 \\ -1 & 0 & 1 \end{matrix}\right]$ | (5) |
| --- | --- |
| $i_{y}=\frac{1}{4}\left[\begin{matrix} 1 & 2 & 1 \\ 0 & 0 & 0 \\ -1 & -2 & -1 \end{matrix}\right]$ | (6) |

Finally, the total gradient is calculated, and the endoscope image when the maximum value occurs is selected, expressed as Eq. (7).

| $S=\sqrt{[i_{x}*\mathrm{I}(x,y)]^{2}+[i_{y}*\mathrm{I}(x,y)]^{2}}$ | (7) |
| --- | --- |

where I is the image, *x* and *y* are the pixel position, is Convolution integral.

***Energy of the Laplacian of the image****
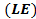
****.*** *The energy of the Laplacian of the image uses the second differentiation of the image intensity function as a high pass filter. Laplace’s operation for searching the larynx boundary in the endoscopic image is used for the second differentiation. The approximately discretized Laplace’s operation kernel is used as a high pass filter. The function for evaluating the endoscopic image quality is defined as Eq. (8):*

| 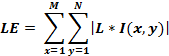 | (8) |
| --- | --- |

where, *M* and *N* are the image size, *L* is the Laplace’s operation kernel, *I* is the image, *x* and *y* are the pixel position and * is the convolution integral.

**Segmentation.** The glottis is a relatively dark region in the image, therefore the gray threshold was used for segmentation^22^. In order to highlight the differences in brightness, keep more details and avoid over-segmentation, this study calculated the gray level of the full image. The lower bound of the threshold of the image was calculated as expressed in Eq. (9). This value was taken as binary threshold.

| 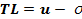 | (9) |
| --- | --- |

where, *u* is the average value and *σ* is the standard deviation.

**Glottis structure discrimination conditions**

(1) Area size

The area was the total number of pixels of the region. Regions that were too small were excluded, and the blocks smaller than 800 pixels were filtered out.

(2) Centroid position

The arytenoid cartilage of vocal cords could be observed only if the glottis was in the image center, therefore regions where the glottis was not in the center were eliminated. Images in which the y-axis has a centroid position lower than 40 or higher than 230 were filtered out.

(3) Aspect ratio

The aspect ratio is defined as the length-width ratio of minimum bounding rectangle of pattern. The glottis shape is an inverted triangle, and east-west regions were filtered out using the aspect ratio. Regions with can aspect ratio larger than 0.9 were filtered out.

**Arytenoid cartilage structure condition**

(1) Area size

The area referred to the total number of pixels of the region. Regions that were too small were eliminated, and blocks smaller than 1000 pixels were removed.

(2) Centroid position

The arytenoid cartilage was located in the upper part of the image, therefore regions where the arytenoid cartilage was not in the upper part were eliminated. Areas where the y-axis had a centroid position lower than 60 or higher than 150 were filtered out.

(3) Aspect ratio

The arytenoid cartilage shape is a rectangular, and the south-north regions were filtered out using the aspect ratio. Regions with an aspect ratio smaller than 1 were filtered out.

**Supplementary Figure 1. BPNN architecture**

|  |  |
| --- | --- |
| |  | | --- |   *X*_1_  *Y*_1_  …  …  …  …  *X*_2_  *Y*_2_  …  …  …  *X*_3_  *Y*_3_  Input layer  Output layer  Hidden layer | |

**Supplementary Figure 2.** Arytenoid cartilage segmentation process: A) gray image, B) binary image, C) arytenoid cartilage segmentation


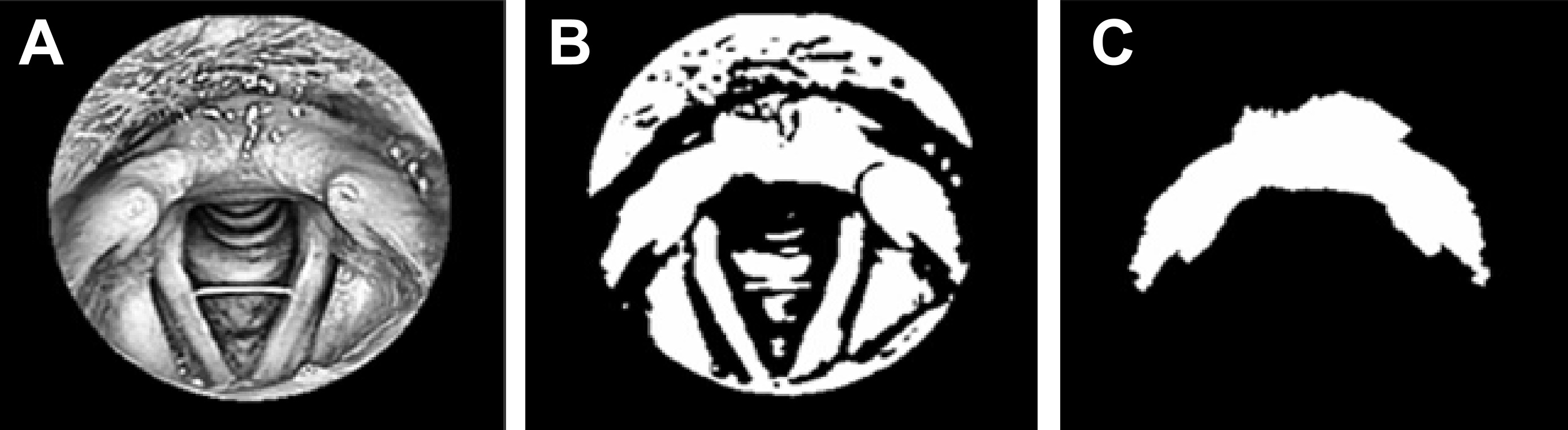


**Supplementary Figure 3.** Vocal cord seed processing: A) glottis image, B) glottis contour, C) vocal cord seed


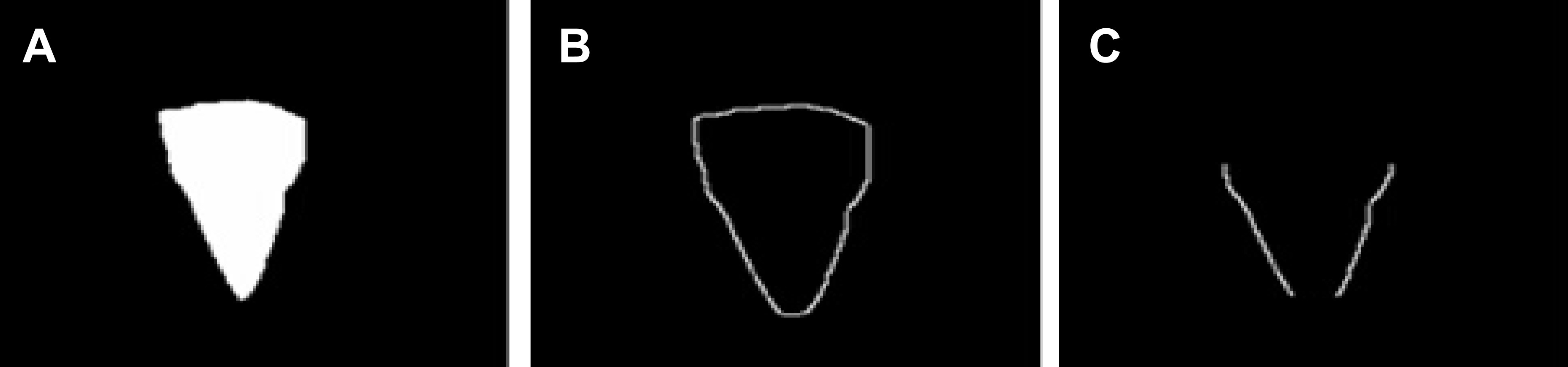


**Supplementary Figure 4.** Adaptive vocal cord segmentation: A) right vocal cord iterated 34 times, left vocal cord iterated 31 times; B) right vocal cord iterated 34 times, left vocal cord iterated 31 times; C) right vocal cord iterated 34 times, left vocal cord iterated 31 times.


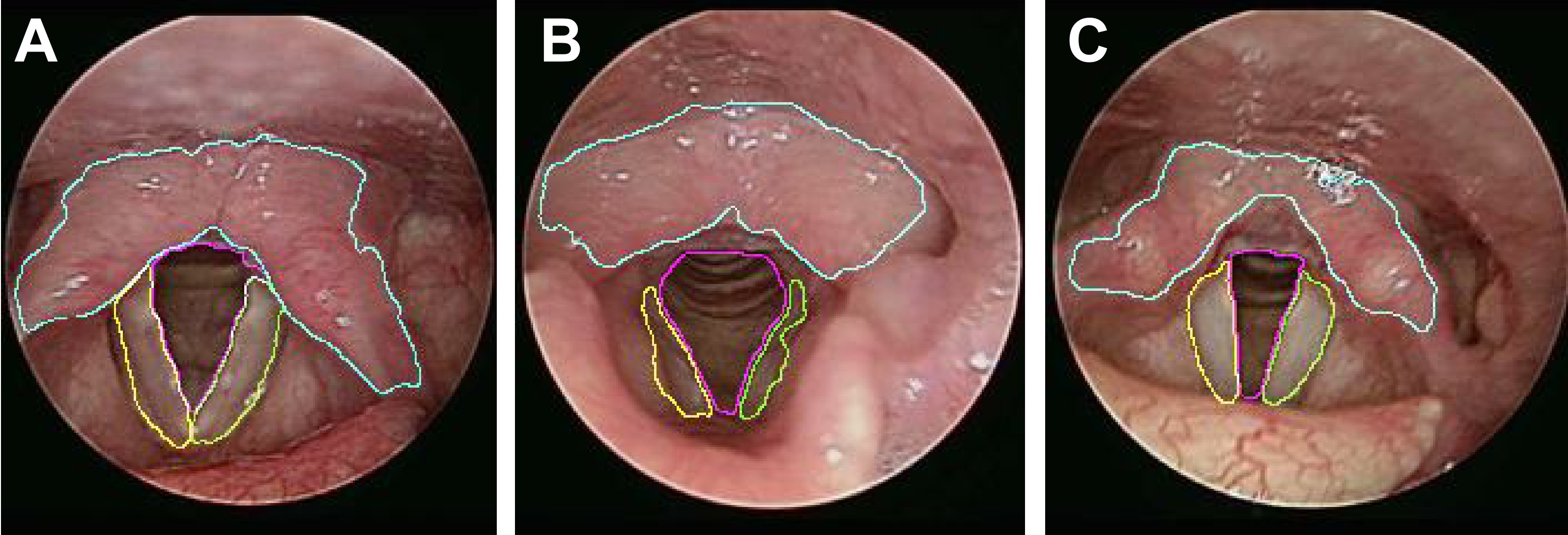

Supplement: Supplementary file 1 — Supplementary file1 (DOC 1258 kb) [file 41598_2020_67587_MOESM1_ESM.doc]
